# Hierarchical forecasting of COVID-19 cases in Africa using machine learning models

**DOI:** 10.3389/fepid.2026.1696282

**Published:** 2026-02-11

**Authors:** Claris Shoko, Caston Sigauke, Katleho Makatjane

**Affiliations:** 1 Department of Statistics, University of Botswana, Gaborone, Botswana; 2 Department of Mathematical and Computational Sciences, University of Venda, Thohoyandou, South Africa; 3 Department of Statistics and Population Studies, University of the Western Cape, Capetown, South Africa

**Keywords:** bottom-up reconciliation, ensemble, hierarchical time series, random forest, weighted average, XGBoost

## Abstract

**Introduction:**

The COVID-19 pandemic posed significant challenges for public health systems, especially in Africa, where data scarcity, inadequate healthcare infrastructure, and regional disparities hindered effective forecasting and response efforts. Conventional forecasting methods have faced challenges in adequately addressing the complexity and detail necessary for effective policy interventions at various administrative levels. This study examines the challenge of producing accurate and coherent forecasts of COVID-19 cases within the hierarchical structure of Africa, which includes the continental, regional, and national levels.

**Methods:**

To establish a comprehensive forecasting model that uses hierarchical time series forecasting through a bottom-up reconciliation approach augmented by machine learning algorithms. We employ extreme gradient boosting (XGBoost) and random forest models, subsequently improving predictive accuracy via a weighted average ensemble method. We produce forecasts at the national level and then aggregate them to ensure consistency across all hierarchical levels. The models are evaluated in comparison to conventional methods such as ARIMA and exponential smoothing.

**Results:**

Empirical findings indicate that XGBoost is the best among all the single forecast models used in this study, combining forecasts from the XGBoost with the random forest and assigning more weights to the XGBoost surpasses all other models in the area of mean absolute error, root mean square error, and mean absolute scale error. Results further revealed that Southern Africa, despite its low population density, reported the highest number of cases, indicating underlying health vulnerabilities and socioeconomic factors. In summary, the bottom-up HTSF method, when combined with machine learning, serves as an effective tool for forecasting in environments with limited data availability.

**Discussion:**

It is advisable to apply similar models to other infectious diseases and to expand their use to guide health interventions, resource allocation, and early warning systems in future pandemics.

## 1 Introduction

The uneven impact of COVID-19 observed throughout the African continent requires careful examination. A combination of underlying socio-economic shortcomings and public health issues has significantly impacted the outcomes of the pandemic. In particular, widespread poverty, limited availability of healthcare services and high rates of infectious diseases such as HIV/AIDS and tuberculosis have likely exacerbated the effects of COVID-19. Evidence from studies, such as that by [1] depicts tuberculosis co-infection associated with increased COVID-19 risk and severity. Decision-making at the operational, tactical, and strategic levels is fundamental for any organisation. Forecasts that inform such decisions possess distinct characteristics. Strategic decisions necessitate long-term forecasts at an aggregate level, whereas decisions at the dynamic operational level demand short-term and detailed forecasts [2]. The variations in time granularity influence the generation of these forecasts; hence, analysts typically produce long-term strategic forecasts by analysing high-level unstructured information from the business environment. Therefore, [3] indicated that short-term operational forecasts are produced using structured but constrained information sources, such as historical COVID-19 data, where this process relies primarily on statistical methods. Moreover, within the same decision-making level, the forecasting literature indicates that varying forecast horizons necessitate distinct methods. This is because these forecasts are generated through different approaches and rely on diverse information sets, leading to potential discrepancies [4], and this disagreement results in misaligned decisions. Daily coronavirus forecasts that inform inventory and health decisions offer a consistent perspective on market stability. Conversely, strategic-level forecasts may indicate a thriving market, which carries profound implications for investment strategies and budgetary decisions [5].

When it comes to time series forecasting, machine learning (ML) outperforms conventional techniques like ARIMA because it can handle large, complex, non-linear datasets, incorporate multiple external factors (features), and continuously adapt with new data. This results in higher accuracy in volatile environments, but it is frequently more complex and less interpretable than simpler statistical models. While ML models like LSTMs and XGBoost capture complex patterns, making them superior for dynamic forecasting, traditional models are excellent for simpler, univariate data but struggle with real-world complexity and huge data. Chenrui et al. [6] explore how machine learning (ML) technologies addressed critical challenges across healthcare and public policy. They argued that ML enables COVID-19 forecasting, diagnosis, and decision support for response and control. In their comprehensive review, [7] analyse how ML has been utilised to combat the COVID-19 pandemic. Bachan et al. [8] summarises how Artificial Intelligence (AI), Machine Learning (ML), and deep learning have expanded medical science’s traditional dynamics during the pandemic in the following key areas.

Hierarchical time series forecasting (HTSF) is essential for better planning, resource allocation, and decision-making because it guarantees coherent (consistent) forecasts across various levels (such as total sales vs. regional sales) in a business structure. It also improves accuracy by utilizing data from both aggregate and granular levels, lowering variance, and effectively managing complex, interconnected data. It resolves the issue of individual estimates not adding up, which leads to inconsistent operations and budgeting, particularly in supply chains, energy, or retail.

The HTSF involves several challenges because the series at various hierarchical levels, reflecting different degrees of aggregation, can display significantly distinct characteristics, as [9] noted. According to [10], supply chain data exhibit intermittency, high volatility, and skewness. In contrast, aggregated series tend to evolve more smoothly, exhibit reduced skewness, and display more pronounced seasonality dependency. The identified differences influence the selection of forecasting methods applicable at various hierarchical levels, and [2] made this observation. We can forecast aggregates by summing the forecasts of the corresponding series at lower levels. This bottom-up approach typically yields suboptimal outcomes, and this situation presents a second major challenge: the necessity for the forecast of each aggregated series to equal the sum of the forecasts of the corresponding disaggregated series, thereby facilitating coherent decision-making across various hierarchical levels. The likelihood of satisfying this aggregation constraint diminishes when forecasts for each series in the hierarchy are generated independently. In many cases, like predicting COVID-19 by the day, the hierarchy can have thousands or even millions of time series at the lowest level, which affects the choice of forecasting methods based on how much computing power is needed.

However, one remarkable aspect of hierarchical forecasting research is the absence of probabilistic prediction, and this is a major drawback, as, in many different contexts, optimum decision-making calls for an evaluation of forecast uncertainty. Gabalawy et al. [11] suggested that to effectively manage forecasts, we need probabilistic forecasts for the total system load in areas like power supply planning, setting operational reserves, price forecasting, and electricity market trading. Although high-level administrative entities, like states or nations, or heavily inhabited regions, frequently have enough data in terms of quantity and quality, this is less prevalent at lower scales and local levels, such as counties. Clustering smaller geographic areas according to characteristics relevant to the COVID-19 spread could help solve this problem by producing clusters with stronger data for subsequent modelling. Using important demographic, mobility, meteorological, medical capacity, and health-related county-level characteristics, [12] suggest a modelling approach that groups countries with comparable epidemic patterns to examine the spread of COVID-19. Building on their work, we provide a hierarchical time series forecasting (HTSF) system meant to forecast COVID-19 cases using these county-level hierarchies while tackling the important issues described before. We think that our proposed HTSF structure, which uses grouped county-level data, can predict COVID-19 cases as accurately as or better than conventional methods for national, cluster, and county-level illness predictions. Our method is especially useful for areas with little or no data.

While machine learning (ML) models (such as Random Forest, and XGBoost) excel in predictive power with large, complex, noisy data, handle high dimensions well, but frequently sacrifice interpretability and require more tuning/data, statistical models (such as ARIMA, ETS) offer interpretability, simplicity for smaller/linear data, and focus on inference but struggle with complexity and scale for HTSF) forecasting [13]. Although specialist hierarchical ML incorporates structure for better results, ML models, particularly deep learning, may capture complex non-linearities and relationships across hierarchical levels more effectively, frequently beating classical statistics in overall accuracy. Table 1 gives a detailed comparison of traditional statistical methods with machine learning applications in HTSF.

**Table 1:** Comparison of traditional statistical models vs. machine learning models.

Feature	Statistical models	Machine learning/DL
Examples	ARIMA, ETS	Random Forest, XGBoost, LSTM, Transformers
Primary goal	Inference and understanding data-generating processes.	Predictive accuracy and pattern discovery in large datasets.
Data nature	Best for small, consistent datasets with linear relationships.	Excels with large, noisy, non-linear, and complex data.
Modeling logic	Series-by-series; often requires manual reconciliation (e.g., Top-Down/Bottom-Up).	Global modeling; can learn hierarchical coherence end-to-end.
Interpretability	High; clear mathematical relationships and variable impact.	Lower; often “black-box” models focused on performance.
Key strength	Reliable for seasonal, low-variance data; requires less data.	Robust to complexity and multi-step forecasting horizons.

We propose a reconciliation method for HTSF utilising county-level data by applying a bottom-up approach, as in the work of [14]. The HTSF method has been effectively utilised in diverse applications, including the prediction of future tourism demand at multiple levels as seen in [15], forecasting demand for accident and emergency departments as in [2], and various business forecasting scenarios as seen in [16]. The HTSF integrates predictions from all aggregation levels to yield temporally reconciled, coherent, and robust forecasts. Hence, in hierarchical forecasting, producing “coherent” forecasts is essential, as the aggregate forecasts must precisely equal the sum of the forecasts at the more detailed and disaggregated levels. This guarantees that the forecasts accurately represent the characteristics of the actual data [17]. Particularly in data-scarce areas at the national and regional levels, this work makes a significant contribution by being the first to utilise bottom-up hierarchical time series forecasting to predict COVID-19 cases in Africa. It shows a new combination of machine learning models, XGBoost and Random Forest—in the HTSF structure, proving their superiority over conventional statistical techniques such as ARIMA (herein referenced as autoregressive integrated moving average) and exponential smoothing in managing complicated and nonlinear epidemic data. The study also suggests a weighted average ensemble (WAE) approach that uses the complementary advantages of XGBoost and Random Forest. Particularly when weighted towards XGBoost, the ensemble method has improved predictive performance, thereby offering a strong option for real-world public health forecasting. Furthermore, the research uses the bottom-up forecast reconciliation technique to guarantee forecast consistency across all levels of the hierarchy, from national to regional to country-level projections. Informed decision-making in public health depends on this consistent forecasting system, which also allows correct resource allocation; hence, the model exposes important geographical differences, such as the high COVID-19 load in Southern Africa despite its comparatively low population density; hence, it stresses the importance of focused interventions. The suggested framework is a useful tool for future epidemic planning and response throughout the African continent, as it is not only scalable and flexible for different infectious illnesses but also provides a realistic forecasting solution in low-data settings.

### 1.1 Research highlights

The highlights of this research are:

- A comparison between traditional hierarchical time series forecasting approaches (ARIMA and Exponential Smoothing) with machine learning approaches (XGBoost and Random Forest)
- Development of a Weighted Average Ensemble model that combines forecasts from the XGBoost and the Random Forest.
- Among the traditional methods, the ARIMA model performed better than the Exponential Smoothing model.
- Machine learning models outperformed traditional statistical methods. Combining machine learning approaches increases the performance of the models in forecasting daily COVID-19 cases.
- Despite having the lowest population density, the Southern African region had the highest COVID-19 prevalence among all the other regions in Africa.

The rest of the paper is organised as follows: Section 2 presents the models, and empirical results are discussed in Section 3. A detailed discussion of the results is presented in Sections 4, 5 concludes.

## 2 Methodology

### 2.1 Data

**Figure 1:**
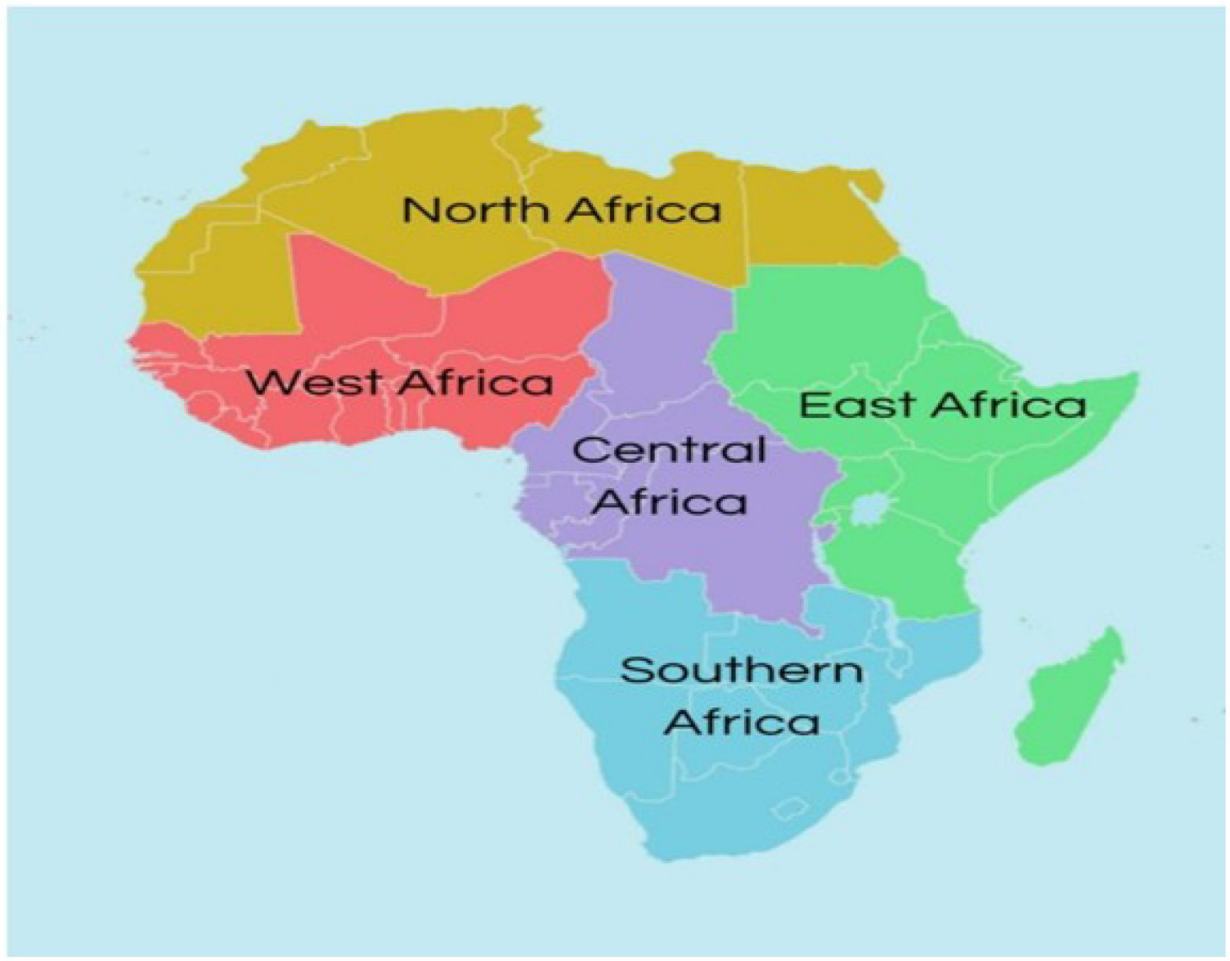
The five regions of Africa.

The data used in this study are daily confirmed COVID-19 cases for the period 2020-02-16 to 2023-02-18 in the five regions of Africa, which are Northern Africa (NA), Eastern Africa (EA), Southern Africa (SA), Central Africa (CA) and Western Africa (WA). The population sizes as of 2024 of each of these five regions are as follows: NA (272 131 339), EA (500 703 846), SA (73 138 701), CA (212 915 636) and WA (456 251 329), both males and females from all age groups. Figure 1 shows the five regions of Africa.

Table 2 provides a classification of African nations divided into five geographical areas: NA, EA, SA, CA and WA. All the regions are further subdivided into their constituent countries, which are numbered for easy identification.

**Table 2:** Regional breakdown of African countries.

Northern Africa	Eastern Africa	Southern Africa	Central Africa	West Africa
1. Egypt	1. Ethiopia	1. South Africa	1. São Tomé and Príncipe	1. Ghana
2. Morocco	2. Kenya	2. Namibia	2. Equatorial Guinea	2. Côte d’Ivoire
3. Algeria	3. Tanzania	3. Botswana	3. Cameroon	3. Guinea-Bissau
4. Tunisia	4. Uganda	4. Lesotho	4. DR Congo	4. Guinea
5. Libya	5. Rwanda	5. Eswatini	5. Republic of Congo	5. Benin
6. Mauritania	6. Eritrea	6. Mozambique	6. Gabon	6. Togo
	7. Somalia	7. Zimbabwe	7. Chad	7. Liberia
	8. Djibouti	8. Zambia	8. CAR	8. Sierra Leone
	9. Sudan	9. Malawi	9. Burundi	9. Mali
	10. South Sudan	10. Angola		10. Niger
	11. Madagascar			11. Burkina Faso
	12. Mauritius			12. Cabo Verde
	13. Seychelles			13. Senegal
	14. Comoros			14. Gambia
				15. Nigeria

### 2.2 Hierarchical structure

A hierarchical time series comprises several time series organised in a hierarchical framework with aggregation constraints that must be met. Let $Y_{t}\in R^{n}$ be the vector of all observations in the hierarchy at the time t, and let $b_{t}\in R^{n_{b}}$ include the most disaggregated (bottom-level) series’ observations. The connection between the whole hierarchy and the base elements can be expressed as Equation 1 below:

$$Y_{t}=Sb_{t},\;\text{for}\;t=1,2,\ldots ,T,$$ (1)

where T is the number of time periods, and S is a summing matrix of hierarchical aggregation rules. Nonetheless, the hierarchical time series aggregation is now given by:

$$Y_{t}=Sb_{t},$$

where $Y_{t}$ is the vector of all series (Africa, regions, and countries) at time t, S is the summing matrix of dimension 60×54 and $b_{t}$ is the vector of bottom-level (country) observations at time t.

$$Y_{t}=Sb_{t}\;⟹\;\left[\begin{array}{c} y_{\text{Africa},t}\\ y_{R1,t}\\ y_{R2,t}\\ y_{R3,t}\\ y_{R4,t}\\ y_{R5,t}\\ y_{c_{1,1},t}\\ \vdots \\ y_{c_{5,15},t} \end{array}\right]=\left[\begin{array}{ccccc} 1_{54}^{T}\\ 1_{6}^{T} & 0_{14}^{T} & 0_{10}^{T} & 0_{9}^{T} & 0_{15}^{T}\\ 0_{6}^{T} & 1_{14}^{T} & 0_{10}^{T} & 0_{9}^{T} & 0_{15}^{T}\\ 0_{6}^{T} & 0_{14}^{T} & 1_{10}^{T} & 0_{9}^{T} & 0_{15}^{T}\\ 0_{6}^{T} & 0_{14}^{T} & 0_{10}^{T} & 1_{9}^{T} & 0_{15}^{T}\\ 0_{6}^{T} & 0_{14}^{T} & 0_{10}^{T} & 0_{9}^{T} & 1_{15}^{T}\\ 5cI_{54} \end{array}\right]\left[\begin{array}{c} y_{c_{1,1},t}\\ y_{c_{1,2},t}\\ \vdots \\ y_{c_{5,15},t} \end{array}\right]$$ (2)

From Equation 2 the summing matrix S row 1 represents the sums of all 54 countries (Africa total), rows 2–6: Sum countries for regions R1 to R5, rows 7–60 denote the identity matrix $I_{54}$ to preserve bottom-level observations with $1_{k}^{T}$ = row vector of k ones and $0_{k}^{T}$ = row vector of k zeros.

**Figure 2:**
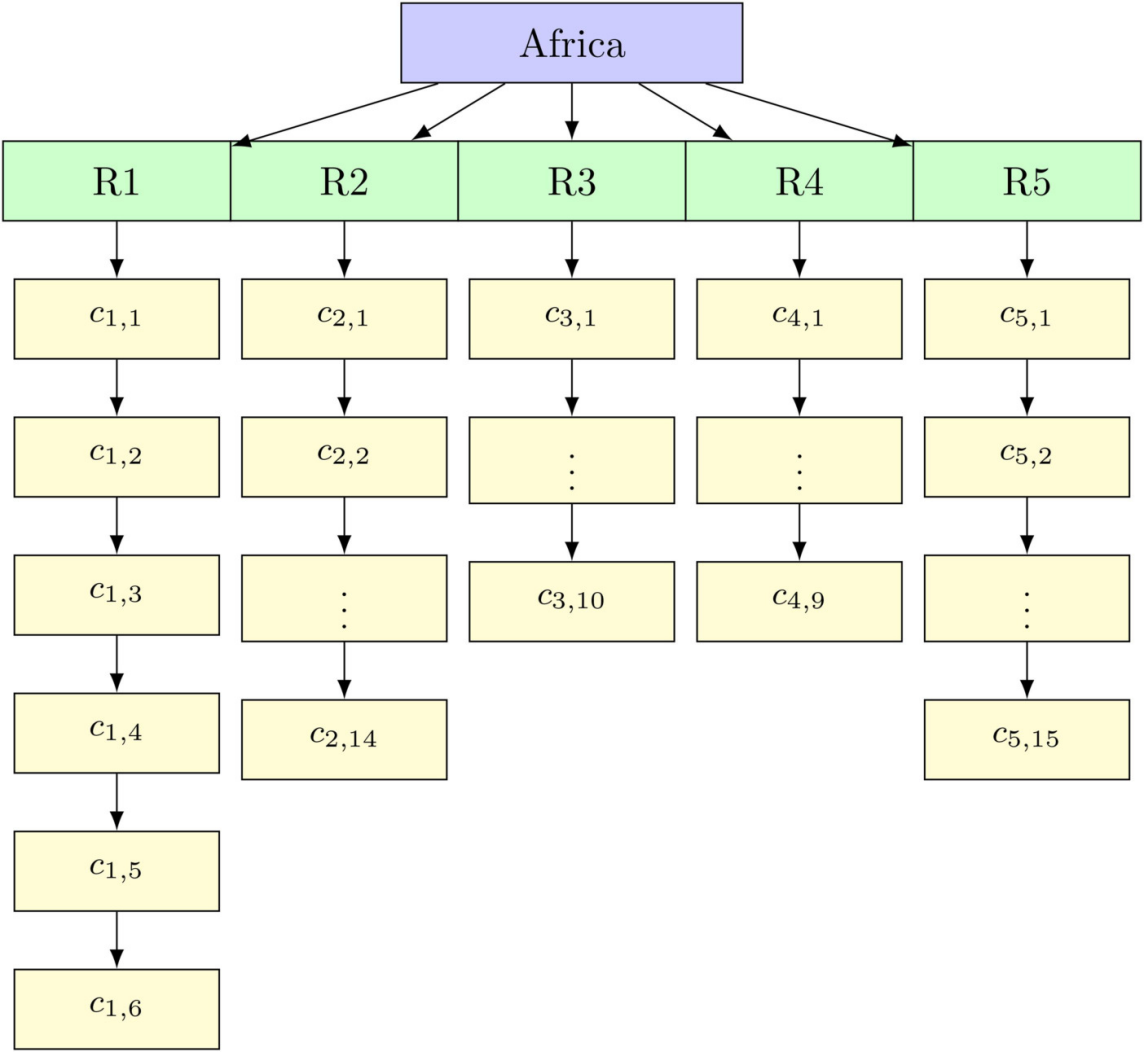
Hierarchical structure of the different countries in the five regions of Africa. The top level is defined as Level 0, Level 1 is represented by the five regions R1(NA),) and R5(WA). The bottom level is Level 2 with $c_{ij}$ denoting country j in region i, e.g., $c_{2,1}$ denotes country 1 in region 2, which is Kenya.

The hierarchical structure of the countries in the five regions of Africa is shown in Figure 2.

### 2.3 Stage 1: machine learning models

This section presents the machine learning reconciliation approach that leverages the potential of decision tree models. The approach is designed to address the limitations of conventional hierarchical forecast methods, such as the ARIMA and Exponential Time Series models, as discussed in Section 1. Given the non-linear nature of COVID-19 daily case data, we use the XGBoost and Random Forest (RF) models. A weighted average ensemble of XGBoost and RF will then be compared with the individual models.

#### 2.3.1 Extreme gradient boosting (XGBoost) method

Extreme Gradient Boosting (XGBoost) is a specific implementation of gradient boosting [18] that incorporates randomisation and regularisation techniques to reduce overfitting while increasing training speed. Moreover, it computes second-order gradients of the loss function, which provide more information about the gradient’s direction, making it easier to minimise the loss.

In an XGBoost framework, weak trees are continuously appended to the set with different weights. The trees in the set must approach the residuals from the previous prediction as closely as possible, as expressed in Equation 3.

$${\hat{y}}_{i}=\sum_{k=1}^{K}f_{k}(x_{i}),\;f_{k}\in F,$$ (3)

where $\hat{y}$ is the predicted value, F is the set including all regression trees, where $f_{k}$ is one of the regression trees, and K is the number of regression trees. The expectation is that the predicted value, ${\hat{y}}_{i}$, is as close as possible to the actual value $y_{i}$ without losing its generalisation ability. The formula to compute Obj as given in Equation 4.

$${\text{Obj}}^{\left(t\right)}=\sum_{i=1}^{n}l(y_{i},{\hat{y}}_{i}^{\left(t\right)})+\sum_{i=1}^{t}\Omega (f_{i})+\text{constant},$$ (4)

where $l(y_{i},{\hat{y}}_{i}^{\left(t\right)})$ is the loss function, representing the influence between the predicted and the true value. The loss function can be a second-order derivative. $\Omega (f_{i})$ is the regularisation term, which defines the complexity of the model. The regularisation term is defined as given in Equation 5.

$$\Omega (f)=\gamma T+\frac{1}{2}\lambda \|\omega {\|}^{2},$$ (5)

where T is the number of nodes, and ω is the score represented by the nodes. The smaller the $\Omega (f_{i})$ value, the lower the complexity and the stronger the generalisation ability.

### 2.4 Random forests

Random Forests (RF) is an ensemble learning approach grounded on decision trees [19], which are statistical models widely utilised for regression and classification problems. The RF methodology, developed by Breiman [20], generates numerous decision trees during training and averages their predictions to improve collective performance [21].

In an RF model, each decision tree is trained on a randomly selected subset of the training data (bagging) and a random subset of features (random subspace method) [22]. There are two important roles that this approach plays: it stops overfitting [23] and enables the model to learn various patterns in the data [24]. Our research focuses particularly on the regression capability of the RF model [25]. Let $X\in X\subset R^{p}$ be the input features, a random vector, and Y∈R be the real-valued response variable. The final goal is to estimate the regression function m(x), which may be defined as the conditional expectation as given in Equation 6 below:

m(x)=E[Y∣X=x]. (6)

Given a training dataset $D_{n}=\{(X_{1},Y_{1}),\ldots ,(X_{n},Y_{n})\}$ comprising n independent and identically distributed observations, we construct an estimator $m_{n}\;:\;X\to R$. This estimator is considered consistent if it satisfies the condition given in Equation 7 as the sample size grows [26]:

$$E[(m_{n}(X)-m(X){)}^{2}]\to 0\;\text{as}\;n\to \infty .$$ (7)

The RF algorithm generates an ensemble of M regression trees. For a given input X, the prediction of the j-th tree can be expressed as Equation 8 according to [27]:

$$m_{n}(X;\Theta_{j},D_{n}),$$ (8)

where $\Theta_{1},\ldots ,\Theta_{M}$ are independent random variables that govern the tree construction process [28]. These variables determine the data subsampling and feature selection at each split. The prediction of the j-th tree takes the form presented in Equation 9 [29]:

$$m_{n}(x;\Theta_{j},D_{n})=\sum_{i\in D_{n}^{*}(\Theta_{j})}\frac{1_{x_{i}\in A_{n}(x;\Theta_{j},D_{n})}Y_{i}}{N_{n}(x;\Theta_{j},D_{n})},$$ (9)

where $D_{n}^{*}(\Theta_{j})$ denotes the bootstrap sample used for constructing the j-th tree, $A_{n}(x;\Theta_{j},D_{n})$ represents the terminal leaf node containing x and $N_{n}(x;\Theta_{j},D_{n})$ counts the number of training points in $A_{n}(x;\Theta_{j},D_{n})$.

The final RF prediction combines the outputs of all individual trees through averaging [30] as presented in Equation 10:

$$m_{M,n}(x;\Theta_{1},\ldots ,\Theta_{M},D_{n})=\frac{1}{M}\sum_{\;j=1}^{M}m_{n}(x;\Theta_{j},D_{n}).$$ (10)

As the number of trees M grows large, we can consider the theoretical infinite forest estimator [31], represented by Equation 11:

$$m_{\infty ,n}(x;D_{n})=E_{\Theta }[m_{n}(x;\Theta ,D_{n})],$$ (11)

where the expectation is taken with respect to the randomness in tree construction, conditional on the training data. The law of large numbers guarantees that as M→∞, the finite forest prediction converges to this infinite ensemble [32], given by Equation 12:

$$\lim_{M\to \infty }m_{M,n}(x;\Theta_{1},\ldots ,\Theta_{M},D_{n})=m_{\infty ,n}(x;D_{n}).$$ (12)

#### 2.4.1 A weighted average ensemble of XGBoost and RF

Ensemble learning combines several machine learning models to achieve better predictive performance than any single model. This study considers the weighted-average ensemble of RF and XGBoost and describes its benefits. The RF and XGBoost ensemble leverages the complementary strengths of bagging and boosting and should yield more accurate and stable predictions than either model alone. A summary of the comparison of the XGBoost model versus the RF model is presented in Table 3.

**Table 3:** Comparison between XGBoost and random forest.

Feature	XGBoost	Random forest
Learning Approach	Boosting (sequential correction of errors)	Bagging (parallel training of independent trees)
Bias-Variance Trade off	Low bias, can over fit if not regularised	Higher bias, but robust to overfitting
Handling Outliers	Sensitive (boosting focuses on errors)	Robust (averaging reduces outlier impact)
Computational Cost	Higher (sequential)	Lower (parallelizable)
Feature Importance	More precise (gradient-based)	Robust but less precise

The XGBoost model excels at optimising complex patterns but can overfit noisy data, while RF provides stability by averaging multiple decision trees. Combining these two models balances the bias-variance trade-off, improving generalisation.

#### 2.4.2 Forecast reconciliation

Hierarchical time series forecasting involves reconciling base forecasts to meet aggregation constraints. Several forecast reconciliation methods are proposed in the literature. Popular techniques include bottom-up (BU), top-down (TD), middle-out (MO), and optimal combination (OC). Bottom-up sums lower-level forecasts, and Top-down aggregates the total forecast. Middle-out is a combination of these. Optimal combination methods, such as ordinary least squares, minimise reconciliation error across the hierarchy, improving accuracy and theoretical consistency. Several other methods are discussed in the literature. For a detailed discussion of these methods, see, for example.

In this study, we will use the BU forecast reconciliation method. Bottom-up methods of probabilistic hierarchical forecasting were introduced by [Taylor et al. [33]]. The Bottom-up (BU) approach is one of the simple methods for generating coherent forecasts. The approach involves generating forecasts for each series at the bottom level and then summing these to produce forecasts for all the series in the structure. The bottom-up (BU) forecasts model is given by:

$${\hat{Y}}_{n}(h)=SG{\hat{Y}}_{n}(h)$$

The BU forecasts are obtained using G=[O|I], where O is a null matrix and **I** is the identity matrix. The matrix G extracts only bottom-level forecasts from ${\hat{Y}}_{n}(h)$, and S adds up to give the BU forecasts.

### 2.5 Performance metrics

The following evaluation metrics will be used in this study.

#### 2.5.1 Mean absolute error

The mean absolute error (MAE) measures the average absolute difference between predicted and actual values, providing a robust metric for model accuracy.

$$\text{MAE}=\frac{1}{n}\sum_{i=1}^{n}|y_{i}-{\hat{y}}_{i}|,$$

where $y_{i}$ is the actual value, ${\hat{y}}_{i}$ is the predicted value and n is the number of observations.

#### 2.5.2 Root mean squared error

The root mean squared error (RMSE) penalises larger errors more severely than MAE, making it sensitive to outliers.

$$\text{RMSE}=\sqrt{\frac{1}{n}\sum_{i=1}^{n}(y_{i}-{\hat{y}}_{i}{)}^{2}}$$

#### 2.5.3 Mean absolute scaled error

The mean absolute scaled error (MASE) compares the model’s MAE to the MAE of a naive (e.g., random walk) forecast. A value <1 indicates better performance than the baseline.

$$\text{MASE}=\frac{\frac{1}{n}\sum_{i=1}^{n}|y_{i}-{\hat{y}}_{i}|}{\frac{1}{n-1}\sum_{i=2}^{n}|y_{i}-y_{i-1}|}$$

#### 2.5.4 Bias

Bias measures the average difference between actual and predicted values, indicating systematic over- or under-prediction.

$$\text{Bias}=\frac{1}{n}\sum_{i=1}^{n}(y_{i}-{\hat{y}}_{i})$$

## 3 Results and analysis

In this section, results from data analysis are presented and interpreted. The process starts with exploratory data analysis, which helps understand the data distributions and visualise the data patterns.

### 3.1 Exploratory data analysis

Table 4 shows that the Southern African region has the smallest population among African regions. In addition, this region has the lowest population density compared to all the other regions. However, the Southern African region recorded the highest daily COVID-19 case count on the African continent. More than 75% of the daily COVID-19 cases in the African continent are from the Southern African region. This could be attributed to challenges related to poverty, access to healthcare, and environmental factors in the region. The Southern African region has a high prevalence of communicable diseases, including HIV/AIDS and TB. HIV/AIDS and TB result in immune deterioration. Hence, people with such infections are at high risk of contracting COVID-19. A study by Tamuzi et al. [1] shows that TB was a risk factor for COVID-19, both in terms of severity and mortality. Although Western has the highest population density, about 3 times that of Southern Africa, the number of daily cases is far less than that of Southern Africa. The Western African region has a lower proportion of the elderly, and some studies have shown that COVID-19 is more prevalent among the elderly than among younger individuals.

**Table 4:** Summary statistics.

	WA	CA	EA	NA	SA	Africa
Population 2024	456,251,329	212,915,636	500,703,846	272,131,339	73,138,701	1,515,140,851
Density (P/${\mathrm{Km}}^{2}$)	75	33	75	35	28	
No. of Countries	15	9	14	6	10	54
Min.	0	0	0	0	0	0
1st Qu.	32	12	154	66	147	849
Median	296	106	924	1,717	1,848	6,137
Mean	668.2	293.3	1,318	3,252	4,673	9,780
3rd Qu.	924	330	1,895	4,269	5,136	13,412
Max.	7,504	9,180	24,523	28,260	54,646	72,463

WA, Western Africa; CA, Central Africa; EA, Eastern Africa; NA, Northern Africa; SA, Southern Africa.

**Figure 3:**
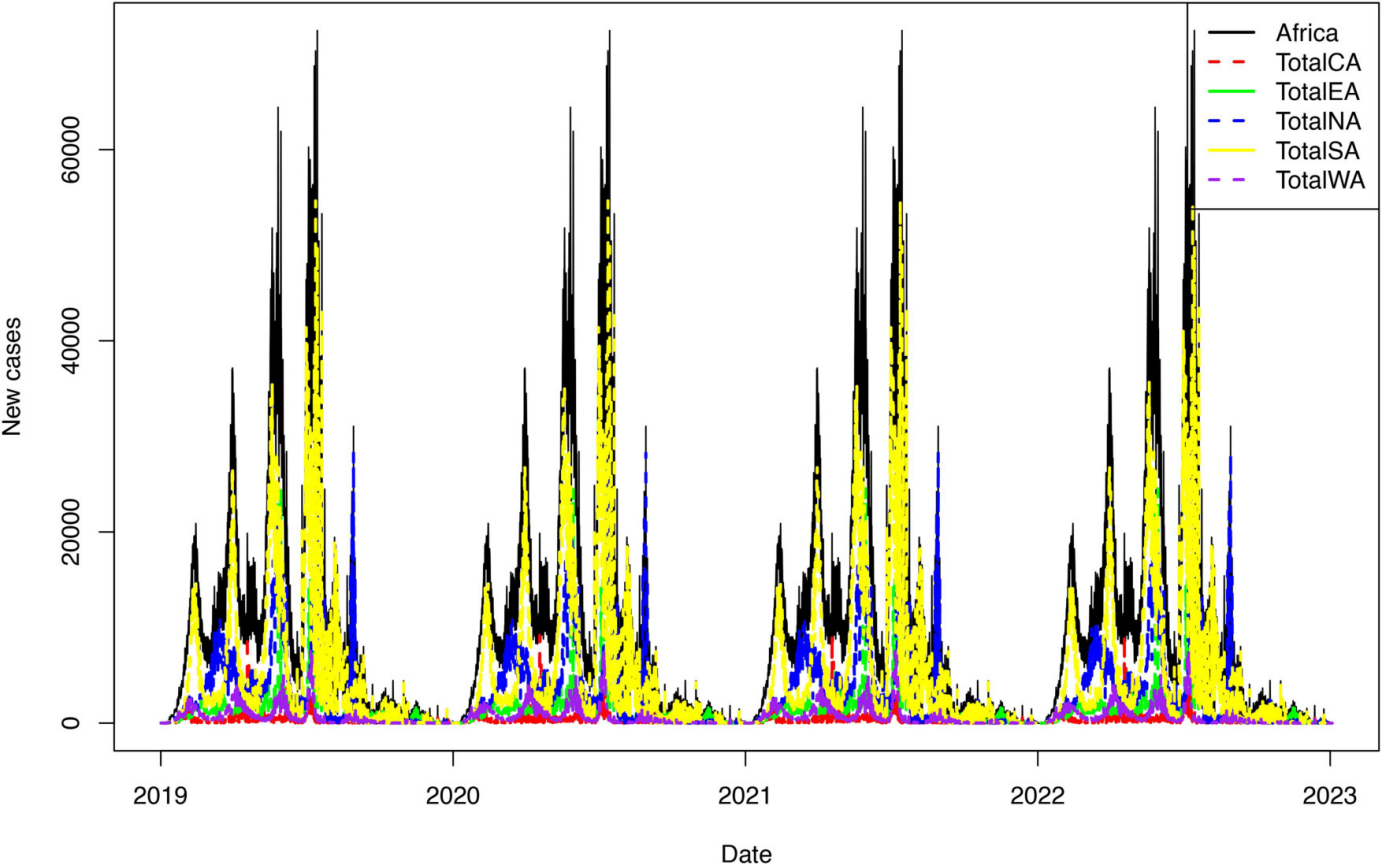
Daily COVID-19 cases for the regions of Africa.

In Figure 3, the time series plots for the daily COVID-19 cases from all the African regions, including the whole of Africa, are presented.

**Figure 4:**
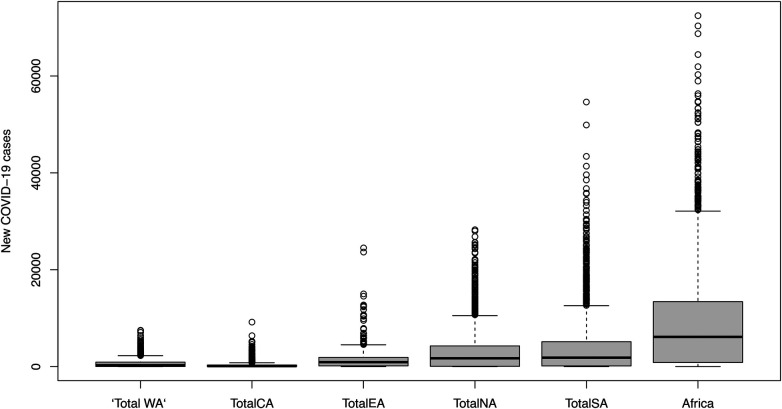
Distribution of new cases by region.

Figure 3 shows that Central Africa had the lowest number of daily COVID-19 cases, followed by Western Africa. Southern Africa accounts for a high share of daily COVID-19 cases reported in Africa. For all regions, the COVID-19 pandemic is characterised by four waves. To examine the distribution of the COVID-19 pandemic, the box and whiskers plots for each region are presented in Figure 4. The Box and Whiskers plots show a non-normal distribution of daily COVID-19 cases with some outliers across the African region. Models that can handle this kind of complex pattern need to be employed. Previous studies have shown that traditional statistical methods, such as ARIMA models, cannot handle such complex data. Thus, machine learning models shall be employed, particularly XGBoost and RF.

**Figure 5:**
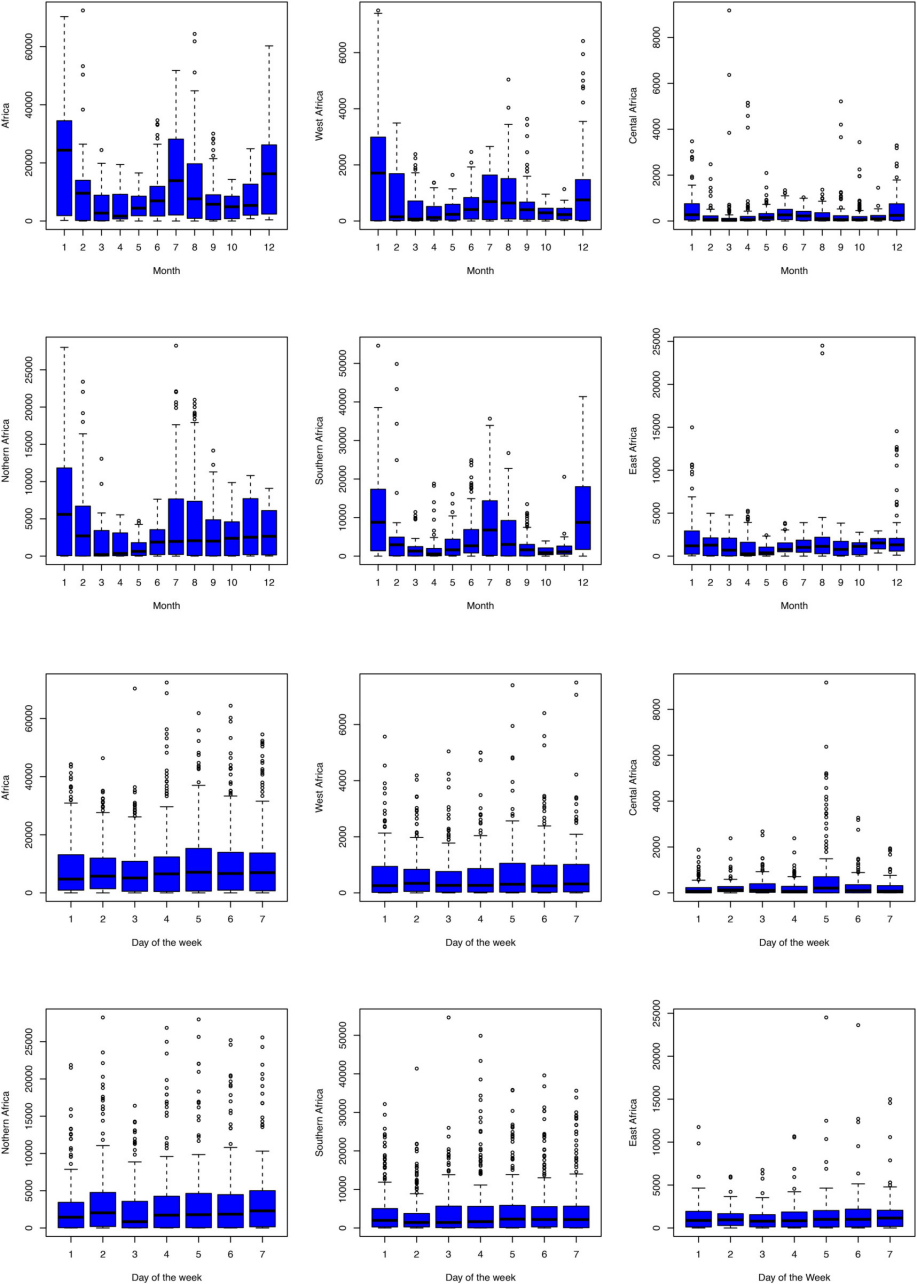
Distribution of daily COVID-19 cases by month and region.

Further distribution of COVID-19 cases by month and day of the week is presented in Figure 5. The results in Figure 5 show that more cases were recorded in winter, from June to August, for all the regions. This is because the coronavirus that causes COVID-19 survives longer in environments with reduced sunlight and lower temperatures. High COVID-19 cases were also recorded around December to January, the months associated with festive seasons, when contact with infected individuals is high, leading to increased spread. Although the distribution of spreads does not vary much by day of the week, Fridays have slightly more reported cases than other days.

### 3.2 Hierarchical forecasting

At this stage, time series data for all African regions had 1,342 observations, which were split into training and test sets at 75:25, yielding 1,000 observations for the training set and 342 for the test set. The machine learning models, XGBoost and the Random Forest (RF), are fitted on the training sets and validated on the test set. To improve the performance of the fitted models, we included lag7 and lag30 variables to capture the effects of week and month on each time series. In addition to lag7 and lag30, the cubic spline for each time series is included. These covariates have a non-zero influence on the daily time series for each region and the whole of Africa.

The hyperparameters were selected from the search ranges provided in Table 5.

**Table 5:** Hyperparameter search space and final values.

Hyperparameter	Search range/values	Description	Final value
Nrounds	{50, 100, 150}	Number of boosting iterations	From bestTune
Max_depth	{1, 2, 3, 4, 5, 6, 7,8,9,10 }	Maximum tree depth	From bestTune
Eta	{0.01, 0.05, 0.1, 0.2, 0.3}	Learning rate	From bestTune
Lambda	{0, 0.1, 0.5, 1, 5}	Minimum loss reduction for split	From bestTune
Colsample_bytree	{0.4, 0.6, 0.8, 1.0}	Column sampling ratio	From bestTune
Min_child_weight	{1, 2, 3, 5, 10}	Minimum sum of instance weight	From bestTune
Subsample	{0.5, 0.6, 0.7, 0.8, 0.9, 1.0}	Row sampling ratio	From bestTune

The final parameters for the XGBoost were tuned using booster = “gbtree,” eta = 0.1, maximum depth = 10, subsample = 0.8, colsample bytree = 0.85, lambda = 1, alpha = 0, objective = “reg:squarederror.”

#### 3.2.1 Extreme gradient boosting (XGBoost) and random forest (RF)

To fit the XGBoost model, the R package “boost” is used. To execute the analysis, three types of parameters were used: (1) general parameters, which in this case are the gbtree that helps to boost, (2) booster parameters, and (3) learning task parameters. To assess performance on the test set, we used the mean absolute squared error (MASE), mean absolute error (MAE), root mean squared error (RMSE), and bias. The forecast performance of the XGBoost for each region is presented in Table 6a. The RF model was fitted using the H2O package. The ones from the fitted RF models are presented in Table 6b.

**Table 6:** Performance comparison of XGBoost and RF models in forecasting COVID-19 spread for African regions.

	(a) XGBoost				(b) RF			
Region	MASE	MAE	RMSE	Bias	MSE	MAE	RMSE	RMSLE
WA	0.4739	13.0261	22.9466	−4.0512	53,642.5	123.899	231.609	0.9096
CA	0.3239	17.6790	23.8315	−14.2687	23,857.88	78.887	154.46	1.12135
EA	0.5808	77.5557	118.7601	−34.1431	298,807.6	200.966	546.633	1.14797
NA	0.5118	41.4553	64.3304	−2.4041	10,42,154	530.6393	1,020.859	1.426211
SA	0.5707	219.8354	377.4192	−112.2297	3,745,006	878.7572	1,935.202	1.614035
AF	0.6840	381.1101	513.4047	−223.8538	87,49,841	1,663.411	2,958.013	1.49935

**Figure 6:**
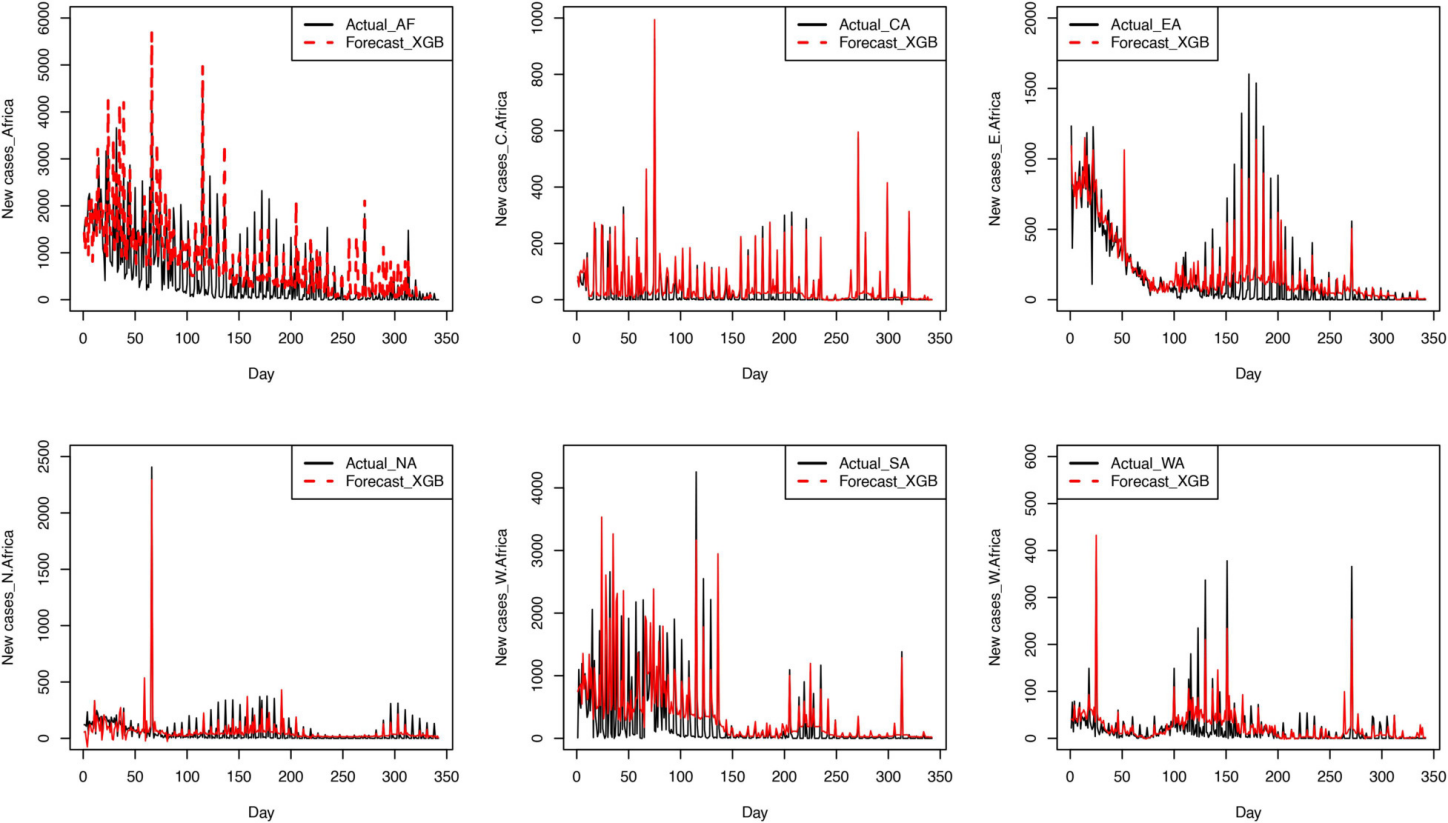
Forecasts from the fitted XGBoost model superimposed on the test sets.

Results from Tables 6a, b show that the MAEs and the RMSEs for the XGBoost model are lower than the ones for the RF model for all the regions of Africa and for Africa as a whole. This shows that the XGBoost model performs much better than the RF model. This finding supports other studies that XGBoost is the best machine learning approach for handling complex relationships due to its built-in regularisation [18]. Figure 6 presents plots of the XGBoost model’s forecasts and observed values.

**Figure 7:**
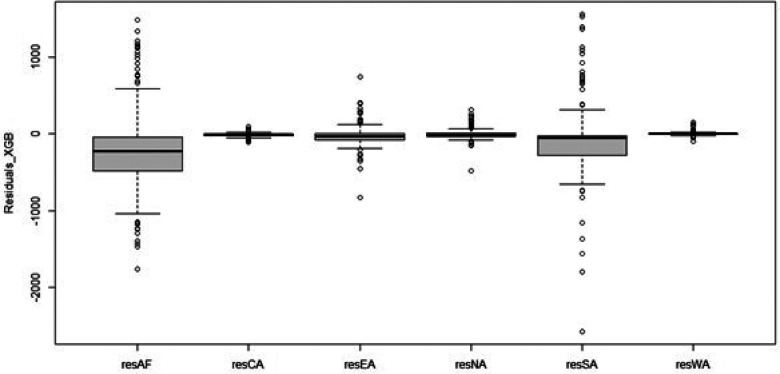
Box and Whiskers plots for the residuals from the fitted XGBoost model.

The plots in Figure 6 show that the forecasts from the XGBoost model are close to the observed test set values. The XGBoost model also predicted most of the spikes in the test set. We also present, in Figure 7, the Box-and-Whisker plots of the residuals from the fitted XGBoost model. For the regions, the plots show the presence of outliers. As such, we propose a proper hierarchical forecasting using the reconciliation procedure, a bottom-up approach, to improve the forecast performance of the models.

#### 3.2.2 Hierarchical forecasting using the bottom-up reconciliation approach

At this stage, hierarchical forecasting is performed using machine learning approaches, including XGBoost and RF models. Forecasts from the XGBoost and the RF are further combined using the weighted average ensemble (WAE), assigning different weights to the fitted models and selecting the model with the best performance. The bottom-up method is used for forecast reconciliation. This results in fitting a two-level hierarchical time-series forecasting model, where Level 1 represents total COVID-19 cases for each of the five African regions, and Level 2 represents daily COVID-19 cases for each country in Africa. The traditional ARIMA and Exponential smoothing are used as benchmarks for hierarchical forecasting.

**Figure 8:**
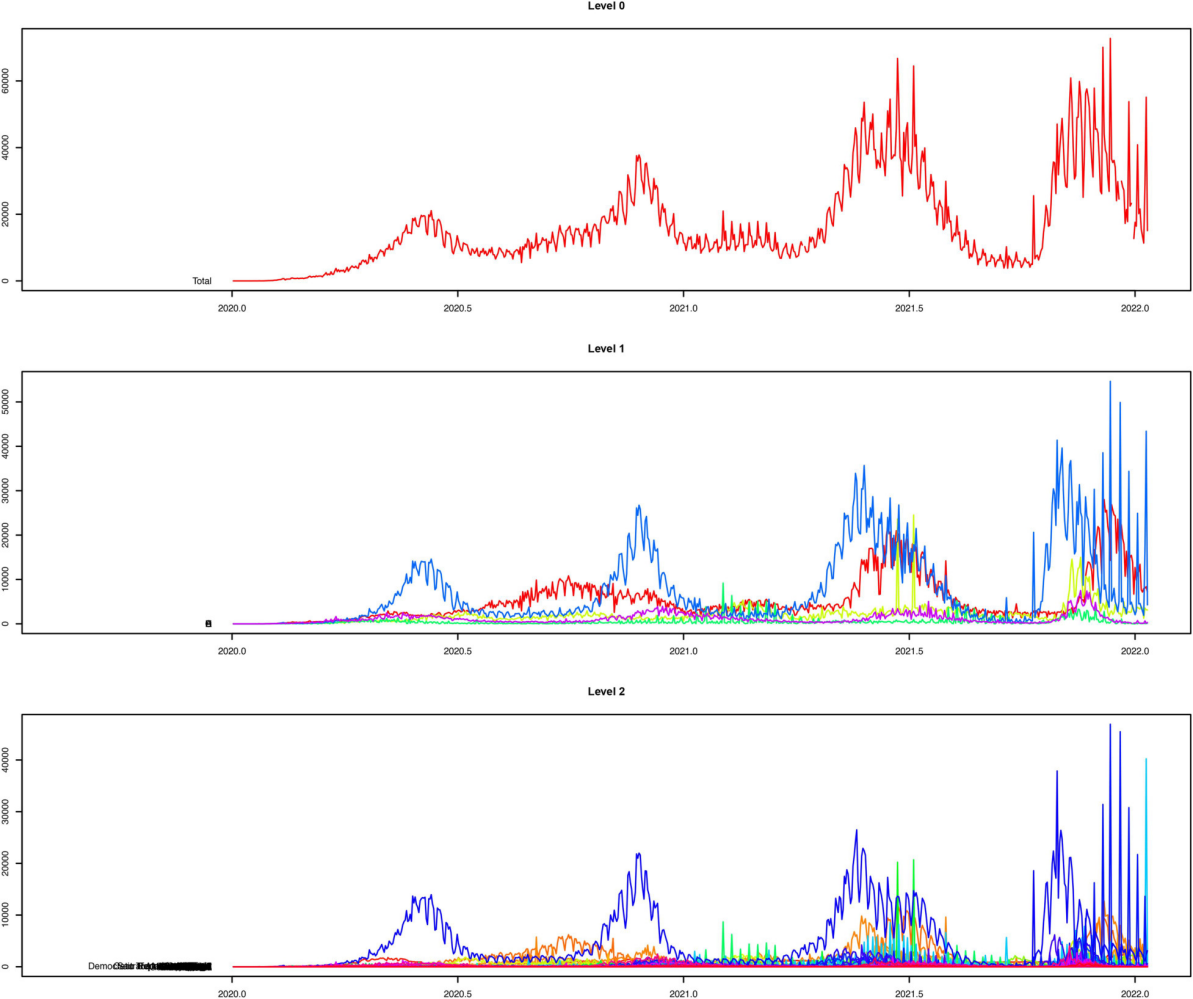
Plots of the reconciled forecasts at all the levels in the hierarchy.

Figure 8 presents the reconciled forecasts for level zero (daily COVID-19 cases in Africa), level 1 (daily COVID-19 cases in the five regions of Africa) and level 2 (daily COVID-19 cases for all countries in Africa). Even though some countries reported more daily COVID-19 cases than others, the time series plots at all levels show a similar trend.

In Table 7, performance metrics (MASE, MAE, and RMSE) for the machine learning approaches (XGBoost, RF, and WAE) and the traditional forecasting methods (ARIMA and Exponential smoothing) are presented.

**Table 7:** Forecasting model performance using the bottom up approach.

Evaluation metric	Africa	NA	EA	CA	SA	WA
Exponential smoothing						
ME	−15,722.2	−5,280.75	−3,211.03	15.51588	−7,094.75	−121.701
RMSE	16,291.29	5,909.292	3,265.748	158.7176	7,702.255	235.6906
MAE	15,846.32	5,678.708	3,211.031	92.81909	7,379.731	202.6874
ARIMA model						
RMSE	22,094.24	5,815.65	3,488.50	162.79	13,835.4049	583.0884726
MAE	20,658.02312	5,554.470786	3,391.231142	117.8476976	11,768.92516	544.8404873
MASE	1.569787	0.9211492	1.612311	0.1957247	1.784315	0.5222791
XGBoost model						
MASE	0.22802	0.1762	0.38977	**0.20111**	0.11121	0.290151
MAE	126.8741	14.27204	51.059	**10.9645**	42.4876	7.9294
RMSE	240.2241	34.51464	116.8128	**48.957**	94.6442	22.26047
RF model						
MASE	0.6930	0.6307	1.1263	0.7364	0.3092	1.3532
MAE	385.5777	51.0857	147.5373	40.1495	118.1217	36.9814
RMSE	599.4324	98.4816	284.9267	168.7576	238.8652	87.1665
WAE model using $0.5F_{\mathrm{XGB}}+0.5F_{\mathrm{RF}}$						
MASE	0.41048	0.3205	0.6687	0.4215	0.1754	0.8561
MAE	228.4037	25.962	87.5959	22.9783	67.0173	23.3972
RMSE	367.9811	50.2951	174.5827	95.295	135.7137	54.2762
WAE model using $0.85F_{\mathrm{XGB}}+0.15F_{\mathrm{RF}}$						
MASE	**0.22243**	**0.1617**	**0.3472**	0.2019	**0.1073**	**0.2504**
MAE	**123.7667**	**13.0978**	**45.4857**	11.008	**40.9884**	**7.5766**
RMSE	**227.4664**	**27.7128**	**101.197**	44.777	**87.9939**	**20.1876**

Bold values represent best performing models for each region.

**Figure 9:**
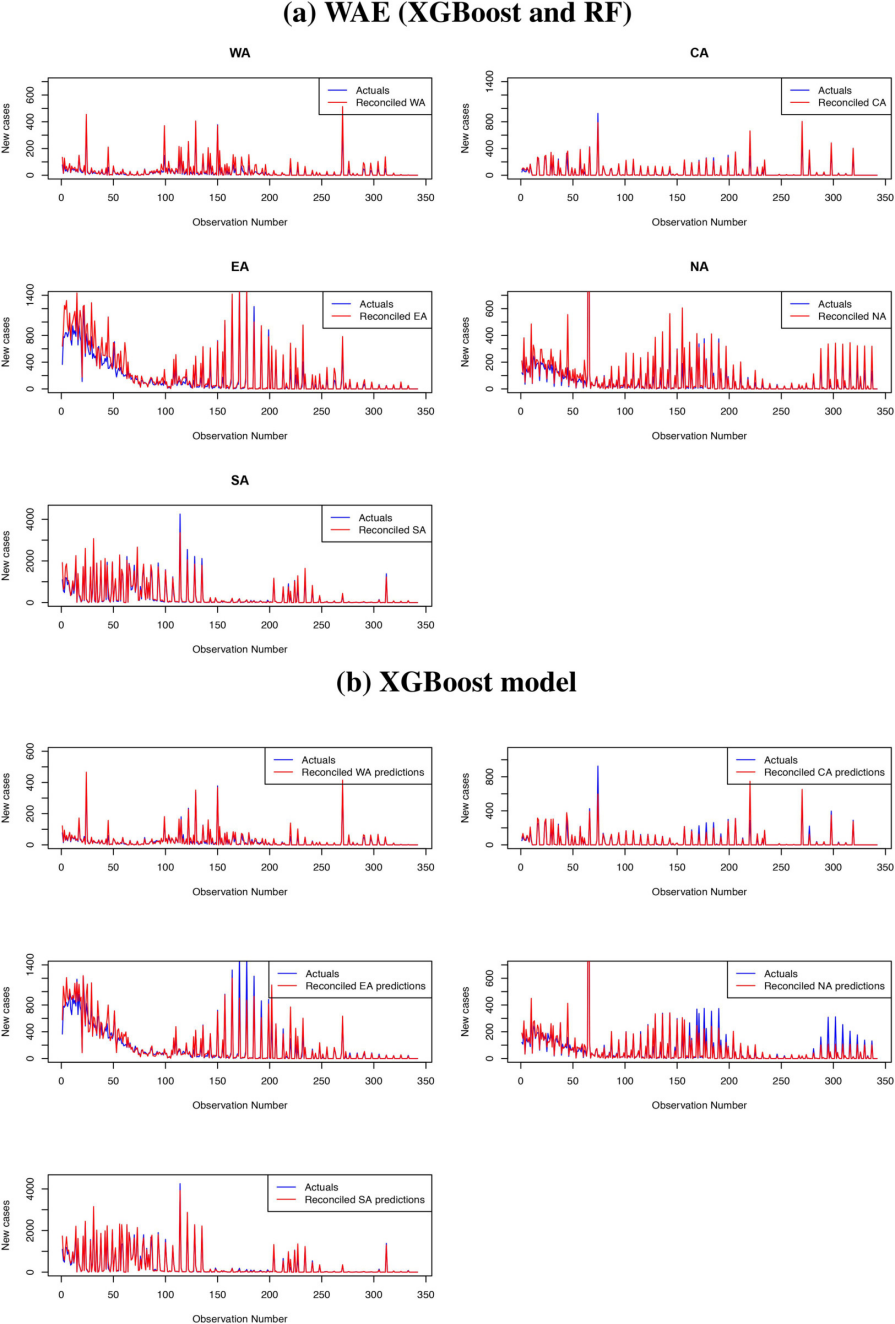
Top panel **(a)**: Hierarchical forecasting from the WAE model for the five regions (level 1). Bottom panel **(b)**: Hierarchical forecasting from the XGBoost model for the five regions (level 1).

**Figure 10:**
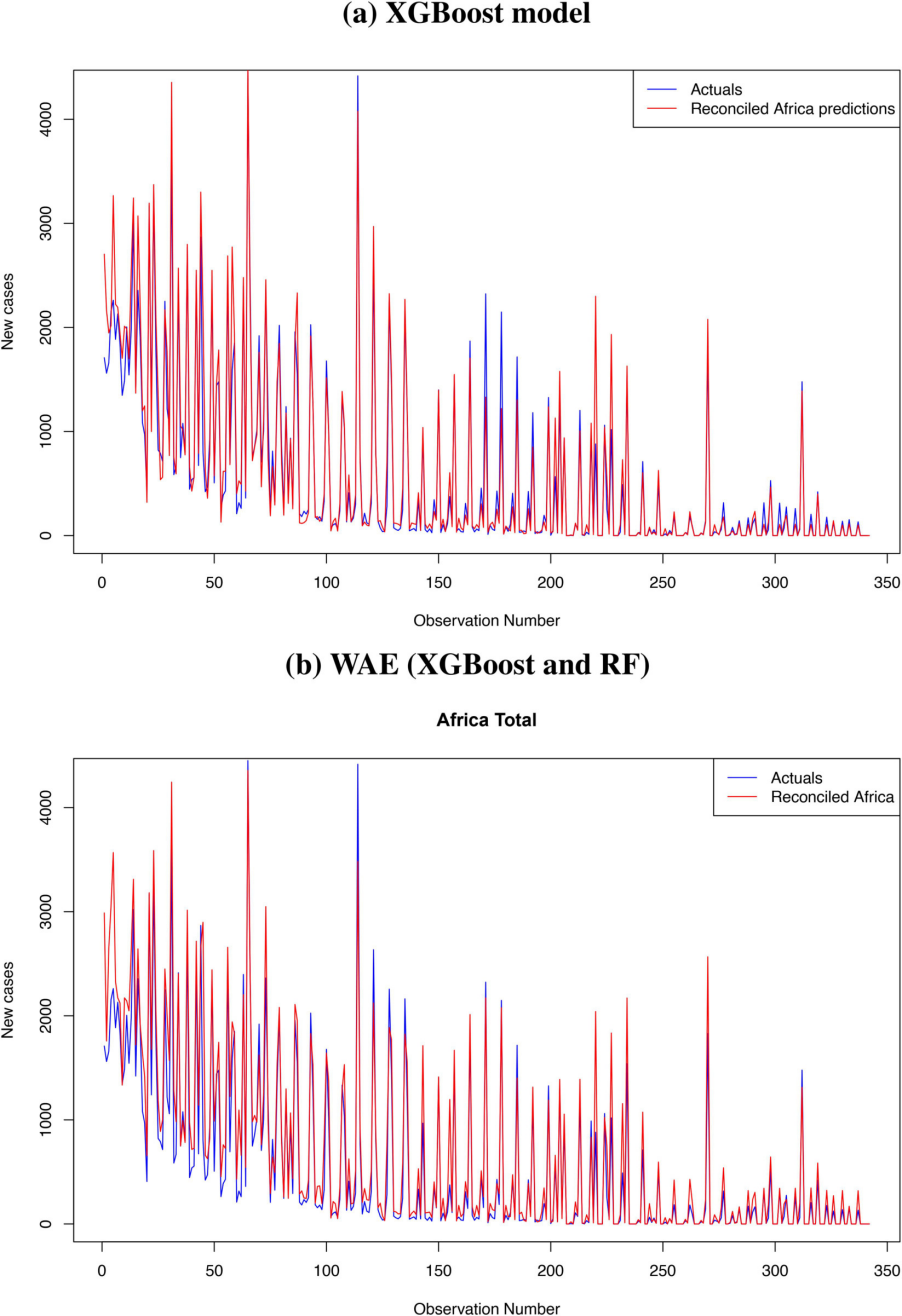
Top panel **(a)**: Forecasts from the XGBoost vs. the observed cases. Bottom panel **(b)**: Forecasts from the WAE vs. the observed cases.

Results from Table 7 show that, among the traditional approaches, the ARIMA model performs better than the Exponential smoothing model. However, the performance of machine learning approaches is far better than that of traditional methods. The XGBoost method has demonstrated greater robustness for hierarchical time series forecasting than the RF and WAE methods, with equal weights assigned to the XGBoost and RF forecasts. The WAE with more weight assigned to the XGBoost forecasts performs better than the one with equal weights, and its metrics, though smaller, are approximately equal to those from the XGBoost method. In addition, the RMSE, MAE, and MASE for the XGBoost hierarchical forecasting using the reconciliation approach are lower than those presented in Table 6 for XGBoost without reconciliation. The same is observed for the RF method, where the performance metrics presented in Table 7 are lower than the ones presented in Table 6. Thus, hierarchical forecasting using the bottom-up reconciliation approach improves forecast accuracy by accounting for the hierarchical structure of the data and forecasting at multiple levels of aggregation. The WAE method with more weights assigned to XGBoost forecasts is the best forecast approach, followed by the XGBoost method. We present plots of forecasts from WAE, with more weight assigned to XGBoost, and of XGBoost forecasts for levels 1 and 0. The plots are presented in Figures 9, 10 for level 1 and the top panel, respectively.

Plots in Figure 9a further confirm that forecasts from the WAE model with more weights assigned to the XGBoost model than the RF give a better prediction of the test set than the forecasts from the XGBoost model presented in Figure 9b. The WAE also give a better prediction of the reconciled COVID-19 cases for the top panel (Africa) than the XGBoost method, as shown in Figures 10a, b, respectively. There has also been a notable improvement in capturing spikes in the datasets when the bottom-up reconciliation approach is used for hierarchical forecasting, as shown in the plots in Figure 6. The coverage probabilities from the XGBoost were estimated using the linear regression approach at a 95% confidence interval and are fairly high. This further confirms the robustness of XGBoost for hierarchical forecasting.

## 4 Discussion

This study compares the performance of machine learning methods (XGBoost and random forests) and traditional methods (exponential smoothing and ARIMA) for hierarchical time-series forecasting of daily COVID-19 cases in Africa. The choice of the XGBoost and the Random Forest machine learning methods is anchored in the study by Shakhovska and others [34], which, although applied to a hierarchical classification problem, supports the selection of the XGBoost and the Random Forest as the best predictive methods. The study used a two-stage approach. In the first stage, we compare the performance of the XGBoost and Random Forest models in predicting COVID-19 cases across the five regions of Africa and in the aggregated time series for the whole African continent. At this stage, reconciliation methods were not used. The XGBoost had lower MASE and RMSE on the validation set than the Random Forest method. Thus, the XGBoost outperformed the Random Forest method in forecasting the daily COVID-19 cases at all levels of the hierarchy. XGBoost’s fine-grained control and ability to handle imbalanced datasets make it a strong contender for advanced predictive modelling [35], unlike other single-forecast models. While XGBoost also has the advantage of incorporating inbuilt regularisation techniques that help prevent overfitting and thereby improve generalisability, the Random Forest does not have built-in regularisation parameters, which can be beneficial in some scenarios [35]. However, based on the comparison of the XGBoost approach across the African regions, West Africa has the lowest MAE, followed by Central Africa, and the North African region is in third place. From the RF approach, Central Africa had the lowest MAE, followed by West Africa, and East Africa came in third. West Africa, Central Africa, and East Africa are the regions with the fewest recorded daily COVID-19 cases. This could explain the small MAEs in these regions.

In the second stage, hierarchical time-series forecasting is performed using the bottom-up reconciliation method. The machine learning approaches are benchmarked against traditional methods, namely ARIMA and Exponential time series methods. A comparison of the traditional time series approaches showed that, overall, the Exponential Smoothing Time series model performed better than the ARIMA model. For the machine learning methods, we used XGBoost and the Random Forest method, and further ensembled them using the Weighted Average Ensemble (WAE). Various weights were assigned to the forecasts from XGBoost and Random Forest, and the results showed that assigning more weight to XGBoost forecasts improved the model’s performance on the validation set. The uniqueness of this study is in the formulation of the WAE approach. Overall, the order of performance of the fitted models can be summarised as follows: WAE: $0.85F_{\mathrm{XGB}}+0.15F_{\mathrm{RF}}>$ XGBoost > WAE: $0.5F_{\mathrm{XGB}}+0.5F_{\mathrm{RF}}>$ RF > ARIMA > EST. Although the XGBoost outperforms the WAE approach, their performance is almost identical. This study shows that, for this problem, the weighted average ensemble (WAE) method with more weights assigned to XGBoost is the best approach for hierarchical forecasting of COVID-19 spread across the African continent. Although a study by Fang and others [36] supports the flexibility of the XGBoost machine learning model for handling time series data with complex patterns like those in the COVID-19 data, combining it with the random forest method improves its efficiency. Thus, combining forecasts improves stability and reduces sensitivity to outliers, leading to more reliable predictions than single-forecast models [37]. A comparison of forecasts from the first and second stages for the XGBoost and RF methods shows that hierarchical time-series forecasting using the bottom-up reconciliation approach improves the predictive power of the models. The bottom-up approach prevents information loss during aggregation and maintains data consistency across all hierarchies [38]. In addition, the approach can capture granular information, leading to more accurate forecasting, especially when there are significant variations across levels. Although in most regions of Africa and across Africa as a whole, the WAE: $0.85F_{\mathrm{XGB}}+0.15F_{\mathrm{RF}}$ performed best, the Central African region showed different results. The XGBosst showed the best performance in the Central African region.

## 5 Conclusion and recommendations

This study shows that a bottom-up approach to hierarchical forecasting can improve the accuracy and consistency of COVID-19 predictions across different levels in Africa. This method provides substantial benefits for public health planning when data availability allows, ensuring that forecasts at national and regional levels align with those at the country level. Machine learning techniques, particularly the weighted average ensemble (WAE) of the XGBoost and RF forecasts, with more weight assigned to the XGBoost, demonstrated greater robustness and accuracy than traditional statistical methods such as ARIMA and exponential smoothing, especially when modelling complex, non-linear epidemiological data.

The study’s key empirical finding indicates that the Southern African region became the epicentre of COVID-19 cases in Africa, despite possessing the lowest population and population density. The unexpected outcome can be attributed to underlying comorbidities, particularly the high prevalence of HIV/AIDS and tuberculosis in the region, which are known to compromise immune response and may have intensified the impact of COVID-19. Future research should investigate the biological and epidemiological interactions among COVID-19, HIV, and TB through in vivo or in vitro studies to enhance understanding of their combined health burden. National health authorities should enhance local data collection and consider implementing combined machine-learning approaches, combined with bottom-up hierarchical forecast reconciliation, to improve pandemic preparedness and response in Africa and other regions with limited data resources.

## Data Availability

The analytic data can be downloaded from https://github.com/.
